# Filamentous ascomycetes fungi as a source of natural pigments

**DOI:** 10.1186/s40694-017-0033-2

**Published:** 2017-05-10

**Authors:** Rebecca Gmoser, Jorge A. Ferreira, Patrik R. Lennartsson, Mohammad J. Taherzadeh

**Affiliations:** 1 Swedish Centre for Resource Recovery, University of Borås, 501 90 Borås, Sweden; 2 University of Borås, Allégatan 1, 503 32 Borås, Sweden

**Keywords:** Pigments, *Neurospora*, Carotenoids, Edible filamentous fungi, Ascomycetes

## Abstract

Filamentous fungi, including the ascomycetes *Monascus*, *Fusarium, Penicillium* and *Neurospora*, are being explored as novel sources of natural pigments with biological functionality for food, feed and cosmetic applications. Such edible fungi can be used in biorefineries for the production of ethanol, animal feed and pigments from waste sources. The present review gathers insights on fungal pigment production covering biosynthetic pathways and stimulatory factors (oxidative stress, light, pH, nitrogen and carbon sources, temperature, co-factors, surfactants, oxygen, tricarboxylic acid intermediates and morphology) in addition to pigment extraction, analysis and identification methods. Pigmentation is commonly regarded as the output of secondary protective mechanisms against oxidative stress and light. Although several studies have examined pigmentation in *Monascus* spp., research gaps exist in the investigation of interactions among factors as well as process development on larger scales under submerged and solid-state fermentation. Currently, research on pigmentation in *Neurospora* spp. is at its infancy, but the increasing interest for biorefineries shows potential for booming research in this area.

## Background

For a long time, filamentous fungi have been used for the industrial production of commercially relevant products, including enzymes, antibiotics, feed products, and many others [1]. The biorefinery concept, i.e., converting substrates to value-added products, is widely accepted within the research community. Therefore, research towards the diversification of established and future facilities for the production of numerous novel and valuable products as well as by-products through fermentation is presently a hot topic. First-generation ethanol plants are good examples where side stream products are utilized to supplement already existing products (e.g., ethanol, animal feed and CO_2_) by producing substances such as organic acids, enzymes, ethanol, biomass for food and/or feed applications, and pigments [1]. In particular, the interest for fermentation-derived pigments in the food and feed industry has increased in recent years [2]. This interest in food-grade pigments is because of the pigments’ ability to enhance the products’ natural color in order to indicate freshness, appearance, safety, and sometimes even to add a novel sensory aspect to attract consumers [3, 4].

**Fig. 1:**
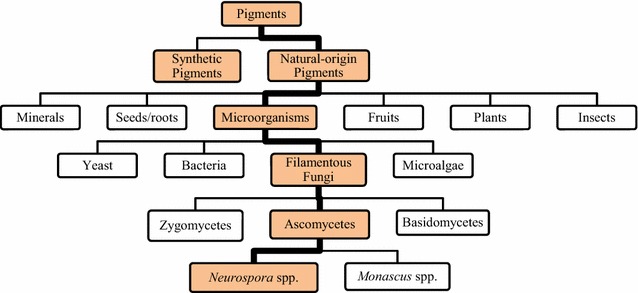
Overview of some sources that can be used for extraction of synthetic or natural-origin pigments [12–14]

Producing pigments from filamentous fungi has great potential [2, 4, 5], not only as an added-value product for biorefineries but also as an alternative to synthetic or other natural pigments that have limitations [6–8]. The increasing demand for pigments of natural origin, particularly in the food sector [9, 10], further increases the interest to investigate filamentous fungi as potential pigment producers. To improve the chances of the pigments and biomass being free from mycotoxins (toxins of fungal origin [11]), particular interest has been taken in edible filamentous fungi that have been used in traditional food products and that can naturally synthesize and secrete pigments. Figure 1 presents an overview of the sources of natural pigments, highlighting the main focus of this review.

A few strains of ascomycetes filamentous fungi being considered as potential pigment producers include, some strains of *Talaromyces* (e.g., *T. purpurogenus* and *T. atroroseus* producing red pigments), *Cordyceps unilateralis* (deep blood red pigment) [15], *Herpotrichia rhodosticta* (orange), *Curvularia lunata* and several species of *Drechslera* (many different pigments). Strains of these species are promising because they are non-mycotoxigenic and non-pathogenic to humans. Nevertheless, the individual mycotoxin profiles of these strains remain to be explored [12]. Some other pigment producing fungi for their use in the production of potential food colorants are species of *Eurotium* and *Fusarium oxysporum* (yellow and red pigments, respectively) [13], *Fusarium fujikuroi* (red [16] and orange pigments [17]) and strains of *Penicillium* [13] such as *P. citrinum*, *P. islandicum* [5], *P. aculeatum* and *P. pinophilum* [5]. However, several species of *Penicillium* are able to produce known toxic metabolites [5] and *Eurotium* spp. and *F. oxysporum* have been shown to produce mycotoxins as well. The potential production of mycotoxins is a major problem which limits the commercial application of these strains of fungi [4]. This problem, together with the increasing demand for natural coloring alternatives from both customers and regulators [4], has triggered investigations and screens for other potential pigment-producing genera of fungi.

After more than two centuries of research, *Neurospora* spp. have been generally recognized as safe (GRAS) [18] with no record of mycotoxin production [1], and thus considered safe for animal and human consumption [19]. This fungus is able to grow rapidly on various types of substrates [20], such as industrial residuals and lignocellulose, to produce ethanol, biomass, and pigments. Particularly, the ascomycete *N. intermedia*, used for preparation of the Indonesian food oncom, has recently been reported as a potential biomass and ethanol producer from waste streams of the industrial process of ethanol production from agricultural grains [1, 21]. However, despite ongoing research and the availability of microorganism-derived pigments, little is known about the production of pigments by *N. intermedia* [6, 9]. Since *Neurospora* species are able to accumulate orange pigments, other species of *Neurospora* such as *Neurospora crassa*, which is genetically and biochemically one of the most well-studied eukaryotic microorganisms [22], have been investigated more extensively regarding the biosynthesis of carotenoids and its regulation [17, 23–25]. *N. crassa* accumulates a mixture of carotenoid pigments such as γ-carotene and neurosporaxanthin [6, 7], the latter carotenoid acid has also been isolated from *Neurospora sitophila* [8].

Several factors have been reported to influence pigment production in ascomycetes [26], although studies on most, if not all, of the mentioned factors are still scarce and superficial. Moreover, even though a great deal of research is available on pigment production by *Monascus* spp. and *Fusarium* spp., information on the performance of the process using both submerged and solid-state fermentation on a larger scale is missing. A deeper understanding and better overview of the factors influencing pigment production, particularly in *Neurospora* as well as in *Monascus* and *Fusarium* spp., is thus desired in order to optimize the process.

The present review gathers available research on pigment production by *Neurospora* species. It identifies main research gaps and consequently provides future research avenues and main challenges towards the use of *Neurospora* spp. for the production of pigments.

## Filamentous ascomycetes fungi as pigment producers

Filamentous ascomycetes fungi are known to produce an extraordinary range of colors. There are a wide selection of non-pathogenic strains of filamentous fungi that are non-toxin producers and can be used as potential sources of natural food colorants with improved functionality [7]. The ability of these fungi to grow on residuals of different complexity (e.g., starch-based, lignocellulose-based residuals) is well-documented, showing versatility regarding different processes that can be built around the ascomycetes fungi [1]. Unlike the use of pigments from vegetables and fruits, the cultivation of ascomycetes does not compete with agricultural land for food production, and therefore, the synthesis of pigments is faster due to time-efficient and simple fermentation processes [7, 27]. The fermentation processes generate high yields of biomass together with value-added products such as pigments, organic acids and alcohols [1].

Regarding pigment production from ascomycetes, even though there is much research on the factors that influence pigmentation in *Monascus* spp. [2, 13, 26, 28–31] and to some extend for *Fusarium* spp. [32–35], the correlations between the factors are still not fully understood [26]. It is likely that a variety of factors with a complex interplay are involved and that they vary among species. Research using *N. intermedia* for pigment production is limited to only a few studies [6, 9, 36]. Moreover, similar to the research with *Monascus*, studies on large-scale production are nonexistent in the literature [26]. Thus, the specific culture conditions that induce pigment production and their properties and the link between different pigments and the level and activity of carotenoid biosynthetic enzymes are not well understood. The physicochemical properties of pigments are further discussed in the review by Priatni [37]. The available information on pigment-producing *Neurospora* spp. has been compiled in Table 1, including fermentation mode and extraction solvent for the produced pigments, along with the pigment concentration.

**Table 1:** Pigment production by *Neurospora* spp. using different experimental set-ups

Ascomycete	Substrate	Mode of operation^b^	Pigment(s)	*λ*_max_ (nm)^a^	Pigment extraction solvent	Production	References
*Neurospora crassa*	60% tapioca by product and 40% tofu waste	SSF	β-Carotene (yellow-orange)	429, 452, 478	THF	295 µg/g	[13, 38, 39]
*Neurospora crassa* 74-OR23-1A	Vogel’s minimal broth	SmF, in 500-mL Erlenmeyer flask	Neurosporaxanthin	477	Acetone	–	[23, 40]
*Neurospora crassa*	Not defined medium with addition of 1% Bacto-Difco agar	Fernbach flasks (200 ml) for 15 days, followed by 1 day in plastic tent (oxygen atmosphere) (SSF)	Neurosporaxanthin	477.5, 504 (in petroleum ether), 471.5, 496 (in acetone)	Acetone, petroleum ether	0.33 mg/g dry weight	[22, 23]
*Neurospora sitophila*	Not defined medium with addition of 1 g/l Bacto-Difco yeast extract	Aerated flasks for 6 days, wet mycelial was then placed on petri dishes in a transparent plastic tent for 1 day. (SSF)	Neurosporaxanthin	477 (petroleum ether)	Methanol, petroleum ether	0.04 mg/g dry weight	[22, 23]
*Neurospora intermedia* N-1	Maltose, peptone, yeast extract, Mg^2+^ fermentationmedia	SmF* in 1 l Erlenmeyer flask	Yellow-orange	480	1 g of spores was extracted with 5 ml of acetone	24.31 µg/g spores	[9]
*Neurospora intermedia* (PTCC 5291)	Vogel’s growth medium	SmF* in 250 ml Erlenmeyer flasks	Mixture of carotenoids	470	50 mg dried mycelia were extracted in 3 ml methanol, re-extracted with 3 ml of acetone	500 mg/l	[36]

^a^Total carotenoids content is determined by spectral absorption at specific wavelengths maxima depending on which solvent the carotenoids are extracted in. The absorbance value correlates to the amount of carotenoids present in the samples

^b^Solid-state fermentation (SSF), submerged fermentation (SmF)

**Fig. 2:**
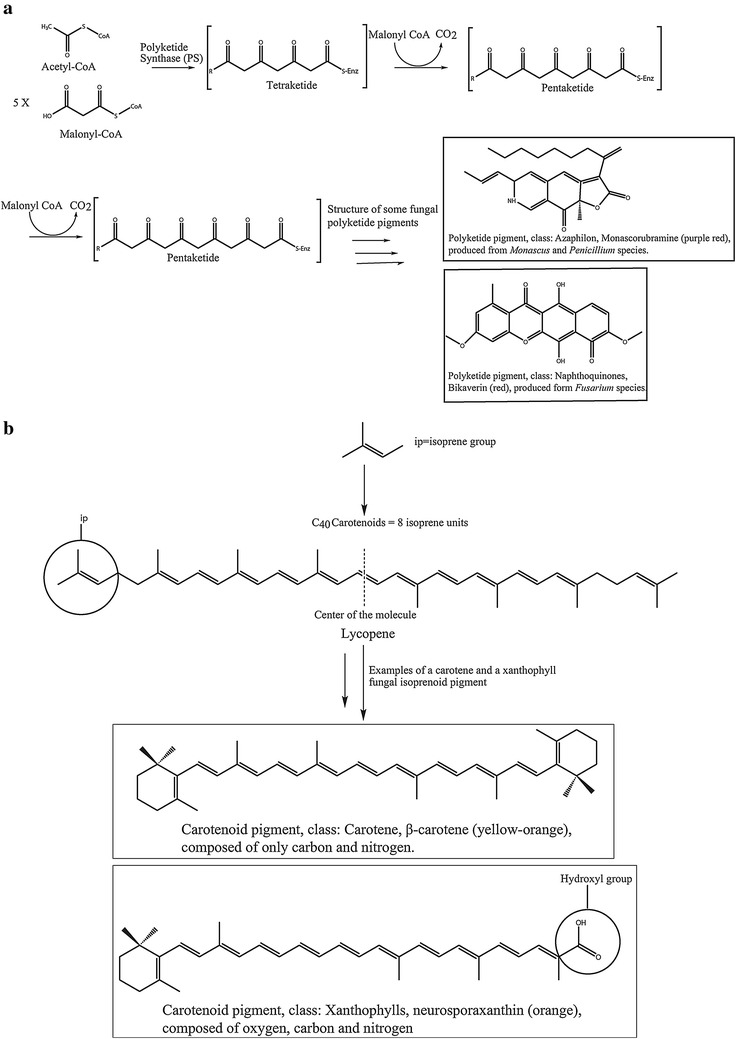
**a** Structure of polyketide pigments followed by two examples of some classes of fungal polyketide pigments. Acetyl-CoA serves as a building block, condensation of acetyl unit with malonyl units and simultaneously decarboxylation result in polycarbonyl compounds that serve as substrates for various cyclases that produce aromatic compounds. **b** General carotenoid structure followed by two examples of a carotene and a xanthophyll carotenoid [5, 44, 45]

The diverse classes of pigments synthesized and secreted by ascomycetes are commonly reported as secondary metabolites [22] of both known and unknown functions [5]. Pigments are generally produced in the cell cytoplasm as a response to disadvantageous environmental conditions, such as nutrient limitation [7, 41], and this process is controlled by a complex regulatory network. Different pigments help to improve fungal survival; for example, carotenoids protect against harmful ultraviolet radiation and light (lethal photooxidation), melanins protect against environmental stress, and flavins serve as cofactors in enzyme catalysis [12]. Pigments produced by filamentous fungi include melanins (dihydroxynaphthalene melanin; a complex aggregate of polyketides), phenazines, flavins (riboflavin), quinones (anthraquinones, naphthaquinones and azaphilones) and carotenoids [2, 12]. Generally, these pigments are chemically classified as either carotenoids or polyketides [5] based on different biosynthetic pathways. *Monascus* spp. pigments are generally produced through the polyketide pathway with some related routes from other pathways such as fatty acid biosynthesis [5, 42], whereas *N. intermedia* pigments are produced through the carotenoid biosynthetic pathway [6]. *Monascus* spp. are usually used as model fungi for polyketide biosynthesis. However, due to the complex pathways involved, there is only rudimentary knowledge about the polyketide pigment regulation. *Fusarium* spp. produces both polyketides (e.g., the polyketide-derived pigment bikaverins) and carotenoids (e.g., neurosporaxanthin) [32]. Polyketides do not seem to be produced in *Neurospora* spp. [43]. Figure 2 illustrates the different pathways involved in polyketide and carotenoid fungal pigment production in broad terms. Carotenoids are yellow to orange-red pigments that are widely used as food colorants. They are produced mainly by microbes belonging to *Myxococcus*, *Streptomyces*, *Mycobacterium*, *Agrobacterium* and *Sulfolobus* [3].

**Fig. 3:**
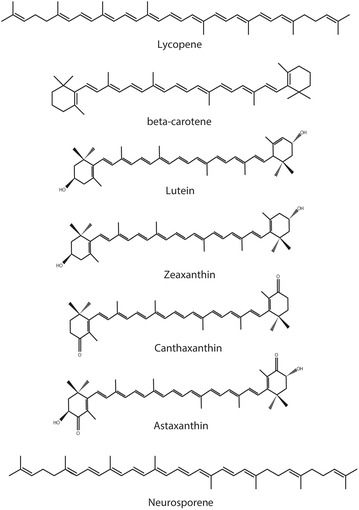
Chemical structures of some common carotenoids found in microorganisms that are also of economic value [8] as well as the structure of the carotenoid acid neurosporaxanthin [22]

Carotenoids belong to the subfamily of isoprenoids, which include a large and diverse class of naturally occurring chemicals [46]. As members of isoprenoids [47], these molecules are derived from eight C_5_ isoprene units and contain 40 carbon units. Based on their molecular structures, carotenoids are therefore further split into two divisions: carotenes and xanthophylls. Carotenes are hydrocarbons, while xanthophylls are derivatives containing oxygen in various functional groups in otherwise similar structures to that of carotenes [24, 48]. Examples of some common carotenoid structures are presented in Fig. 3 [14, 25, 37].

Most of the *Neurospora* spp. have been identified in tropical and subtropical areas in the world and, to some extent, in temperate areas of western North America and Europe as well [49, 50]. Five species of *Neurospora* were identified in Europe, namely, *N. crassa, N. discreta, N. intermedia, N. sitophila* and *N. tetrasperma*. These strains are similar in morphology, and the color of their conidia are orange or yellow-orange caused by the different types of carotenoids. *N. crassa* is the most well-known. Most attempts to increase its carotenoid production has been carried out through photoinduction [51, 52]. Since the carotenoid biosynthetic pathway of *N. intermedia* is comparable to that of *N. crassa* [53] and *F. fujikuroi* [14, 54], studies using *these fungi* are also of interest.

## Carotenoids

Carotenoids are among the most common of all natural pigments on the market [46], and among them, β-carotene, lycopene, astaxanthin, canthaxanthin, lutein, and capxanthin have been exploited commercially (Fig. 3) [8]. Carotenoids are usually extracted from carrots, citrus peels, tomatoes, and algae [6]. Bacteria and carotenogenic species of fungi can also produce them. It is the conjugated double bond system in the carotenoid structure that acts as the chromophore for wavelength-selective (light) absorption, giving these compounds an attractive bright yellow to red color. The absorbed energy leads to electron excitation and changes in orbital occupancy, local bonding, and charge distribution. The non-absorbed light is transmitted and/or reflected to be captured by the eye [44]. Each desaturated reaction shifts the absorption maxima towards longer wavelengths resulting in different yellow to red colors of carotenoids [14]. The double bonds in the carotenoid structure can exist in *trans* and *cis*, but in nature, they are generally in all-*trans* form [55]. The transformation from *trans* to *cis* can be done by acid, heat, oxygen and exposure to light [56]. Nevertheless, the shift from an all-*trans* arrangement to *cis* only results in a minor loss in color strength and hue [55].

Similar to other metabolites, carotenoids have ecological functions and are of value to the fungi. For example, as mentioned before, they can protect against lethal photooxidation [12]. Sterols, dolichols, and ubiquinones fulfill essential cell functions, while secondary carotenoids, such as astaxanthin and canthaxanthin, are accumulated as a response to environmental stress [46]. For example, they can be integrated into the cell membrane to improve its fluidity under high or low temperatures, high light conditions, or when the lipids become more unsaturated. Furthermore, carotenoids serve as precursors of several physiologically important compounds in fungi, such as apocarotenoids (e.g., the fungal pheromone trisporic acid), which are synthesized through the oxidative cleavage of carotenoids. *Neurospora* is one of the carotenogenic genus of fungi, producing a mixture of carotenoid and apocarotenoid pigments. The major component of the carotenoids produced by *Neurospora* is the C_35_-apocarotenic acid, neurosporaxanthin (see Fig. 3) [14, 47]. The closely related filamentous fungus *F. fujikuroi* is also known to synthesis neurosporaxanthin, and have contributed extensively to a better understanding of the neurosporaxanthin pathway and its regulation [35, 57].

### Carotenoid biosynthesis

There is a limited amount of knowledge on the regulation of carotenoid biosynthesis, accumulation, and storage in filamentous fungi. Studies covering this area have only resulted in hypotheses and further research to explain the regulation on a cellular level is required. It has been proposed that genes encoding enzymes involved in isoprenoid and carotenoid biosynthesis are subjected to positive and negative feedback regulatory processes. Several of these molecules are able to mediate signaling processes in order to attain a balanced supply of precursors and assure the adjustment of biosynthesis in response to developmental and environmental cues [58]. It has been hypothesized that the genes are silent under optimal culture conditions and only activated under certain conditions [13]. Even though the molecular mechanisms are awaiting a more detailed understanding, the carotenoid biosynthesis process is undoubtedly influenced by culture conditions, and can therefore be optimized [13].

It is known that filamentous fungi are able to sense and respond to external signals, such as environmental stress. Heterotrimeric G proteins (G proteins) are key signaling elements that communicate external signals to the cells, triggering the upstream regulation of fungal secondary metabolites (e.g., carotenoids) as a response [59]. The levels and activities of carotenoid biosynthetic enzymes and the total carbon flux through the synthesizing system are also important stimuli [46]. These carotenoid molecules are hydrophobic, and they can generally be found in lipid globules and the endoplasmic reticulum membranes. Accordingly, the enzymes involved in carotenogenesis are membrane-bound. However, the regulatory mechanisms controlling the cell compartment where carotenoids are synthesized or stored are still unknown for the *Neurospora* spp. [14]. Structural properties and biosynthesis aspects of carotenoids focusing on *Neurospora* spp. are available in a review by Priatni [37].

**Fig. 4:**
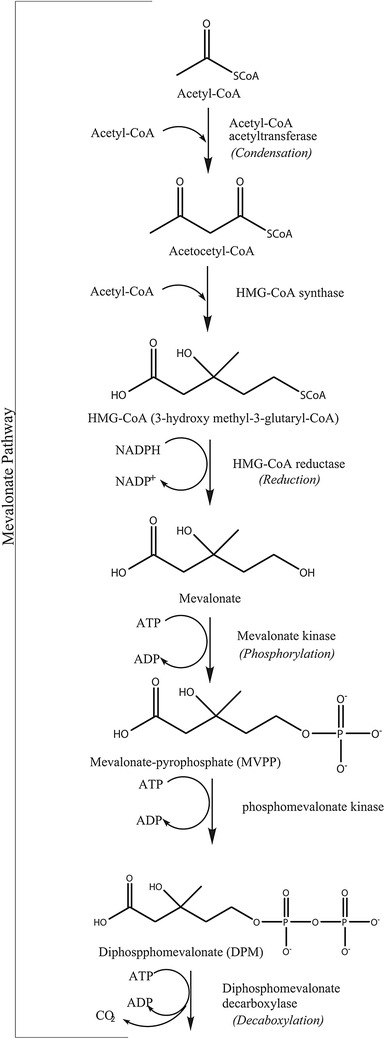
The possible carotenoid biosynthetic pathway of *Neurospora crassa.* The gene products/enzymes responsible for each enzymatic reaction are indicated. Site of chemical changes from precursor molecules are *shaded*. Molecular groups that distinguish xanthophylls from carotenes are marked with *red circles*. The pigments found in *N. intermedia* N-1 are presented in *boxes*

**Fig. 5:**
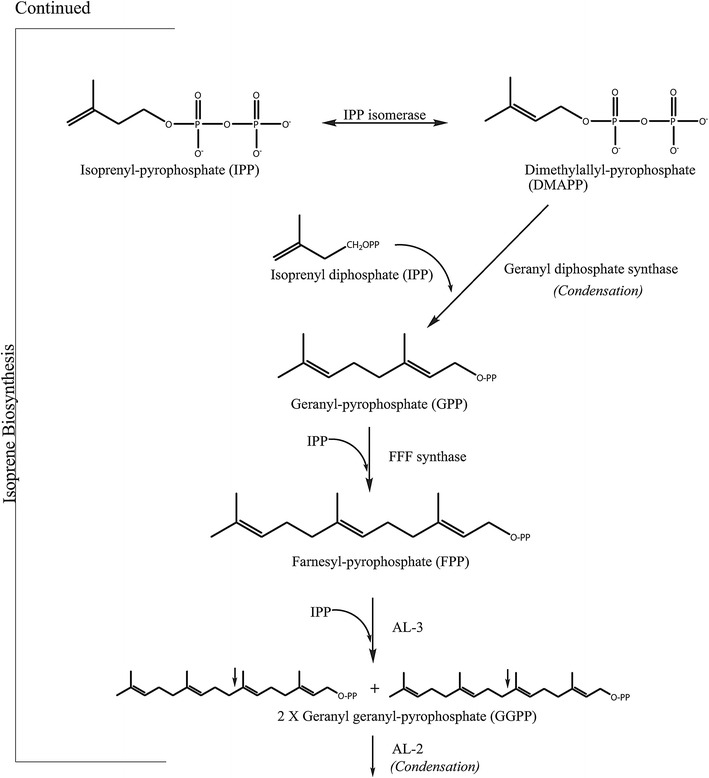
The possible carotenoid biosynthetic pathway of *Neurospora crassa.* The gene products/enzymes responsible for each enzymatic reaction are indicated. Site of chemical changes from precursor molecules are *shaded*. Molecular groups that distinguish xanthophylls from carotenes are marked with *red circles*. The pigments found in *N. intermedia* N-1 are presented in *boxes*

**Fig. 6:**
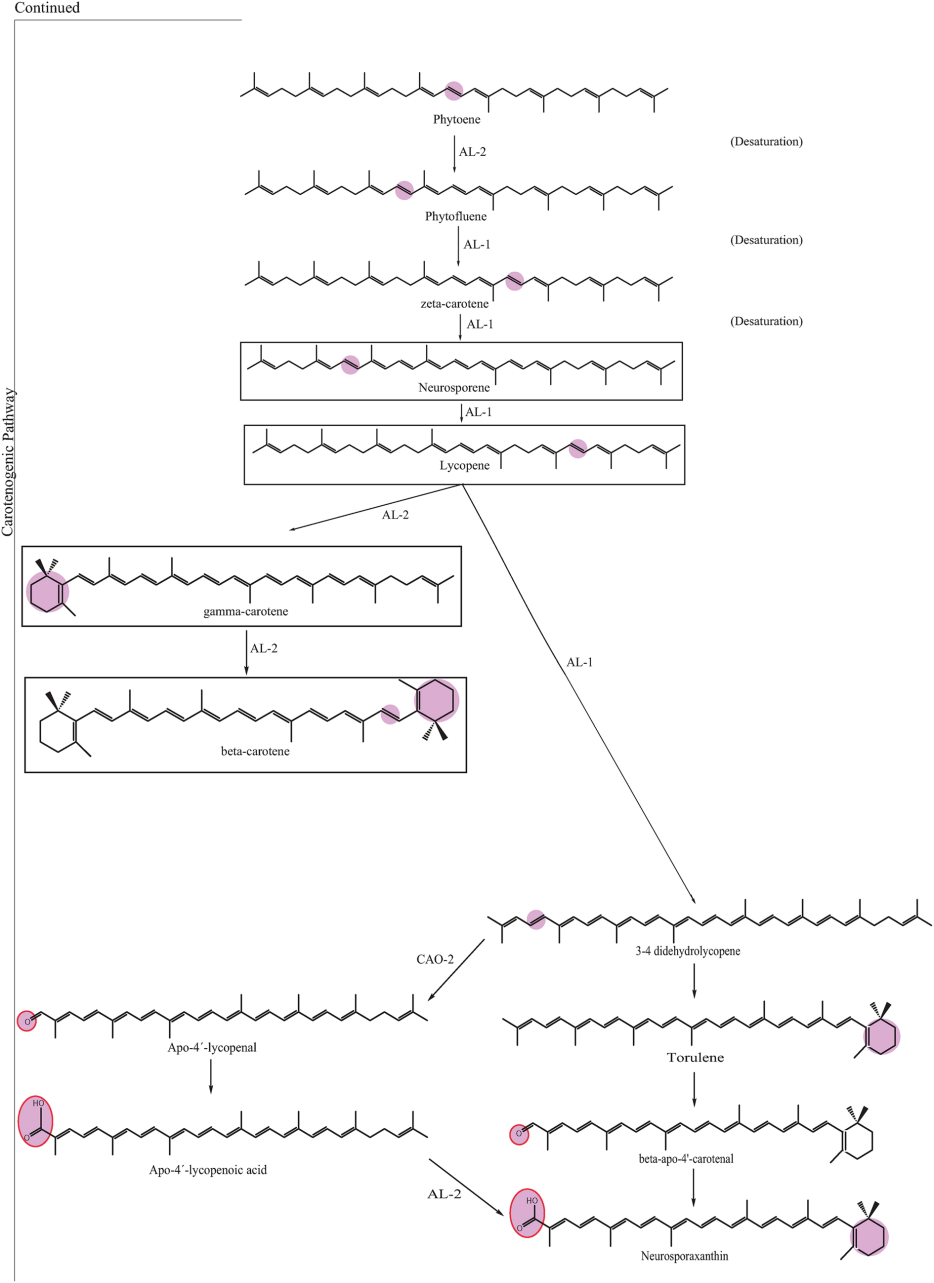
The possible carotenoid biosynthetic pathway of *Neurospora crassa.* The gene products/enzymes responsible for each enzymatic reaction are indicated. Site of chemical changes from precursor molecules are *shaded*. Molecular groups that distinguish xanthophylls from carotenes are marked with *red circles*. The pigments found in *N. intermedia* N-1 are presented in *boxes*

 The genes encoding the enzymes involved in carotenoid biosynthesis have been biochemically investigated [14]. *N. crassa* have been used, along with *F. fujikuroi* that has a similar carotenoid pathway as *N. crassa* [60], as a research model to investigate the carotenoid biosynthesis and researcher have made discoveries on the regulation of pigment biosynthesis by using *Fusarium* species [61]. Acetyl-CoA has been suggested to have an impact on the production of secondary metabolites, such as pigments, by being a primary precursor, depending on the available enzyme pool [59]. Figures 4, 5 and 6 show the carotenoid biosynthetic pathway of *Neurospora* spp. initiated via the mevalonate pathway that leads to the synthesis of short, five-carbon isoprenoid precursors. The isoprenoid biosynthesis pathway is common for all carotenoids. In addition, the last steps in Figs. 4, 5 and 6 present the carotenogenic pathway in *N. crassa*. It has been suggested that the biosynthesis pathway in *N. intermedia* is similar to that in *N. crassa* [37, 53, 62]. The carotenoid pathway is initiated by the condensation of two geranylgeranyl pyrophosphate (GGPP) molecules by the bifunctional enzyme with both phytoene synthase and lycopene cyclase activity, *al*-*2* of *N. crassa*, to produce the colorless carotene–phytoene, the precursor to different carotenoids. The corresponding enzyme in *F. fujikuroi* is named *CarRA*. Phytoene desaturase, encoded by phytoene dehydrogenases *al*-*1* in *N. crassa* (*CarB* in *F. fujikuroi*) (Figs. 4, 5 and 6), mediates the introduction of up to five conjugated double bonds into a phytoene backbone to produce dehydrogenated colored carotenoids [14]. The carotenoids are then subjected to one or two cyclization reactions by cyclases, which introduce α- or β-ionone rings at one or both ends of the polyene chain [63]. The action of *al*-*1* and *CarB* results in different colored intermediates, namely, 3,4-didehydrolycopene, ζ-carotene, neurosporene, lycopene and β-carotene. 3,4-Didehydrolycopene is synthesized by lycopene cyclase to yield the reddish carotene torulene. One cleavage reaction and two oxidation steps are then required to produce the final apocarotenoid neurosporaxanthin in *Neurospora* from torulene. First, torulene is converted into β-apo-4-carotenal by the torulene-cleaving oxygenase *Cao*-*2* in *N. crassa* or *CarT* in *F. fujikuroi*. It was the identification of *carT* that led to the identification of its *Neurospora* orthologue, *cao*-*2*. Next, β-apo-4′-carotenal is further oxidized to neurosporaxanthin by the aldehyde dehydrogenase *ylo*-*1* in *N. crassa* and *CarD* in *F. fujikuroi* [14, 54]. In summary, the five genes needed to produce neurosporaxanthin in *N. crassa* are *al*-*3, al*-*2, al*-*1, cao*-*2* and *ylo*-*1.* Thus, the order of reactions in the biosynthesis and, therefore, the type of carotenoids produced depends on the degree of oxidative stress [35] or temperature of growth [14, 37, 47], among other things. Singgih Marlia, et al. [9] evaluated the carotenogenesis of *N. intermedia* N-1 in a liquid fermentation system and were able to identify five carotenoid compounds in the spores, namely lycopene, neurosporene, γ-carotene, β-carotene and phytoene [9]. Table 2 includes the structures and colors of carotenoid compounds found in *N. intermedia* N-1.

**Table 2:** Structures, application areas and color of the carotenoid compounds found in *Neurospora intermedia* N-1 namely lycopene, neurosporen, γ-caroten, β-carotene and phytoene

Carotenoids	Structure	Applications	Color
Lycopene		High antioxidative activity [64] with beneficial effects on health by fighting many diseases [65]. Used in nutraceuticals and related applications [66]. Used for food coloring (E160d) [67]	Dark red [7]
Neurosporen		Antioxidative properties [68]. Data not found on industrial applications	Yellow-orange [68]
γ-Carotene		Data not found on industrial applications	Orange red [7]
β-Carotene		Used for food coloring (E160a) [67]. Beneficial effects on health by fighting many diseases [65]	Yellow to Orange [7]
Phytoene		Key carotenoid intermediate as a precursor to other carotenoids	Colorless [69]

Factors that have been previously reported to influence pigment production in other strains can thus be considered as potentially important in other filamentous fungi as well. Based on already existing processes and previous knowledge of carotenoid production pathway in filamentous fungi, these factors are individually discussed in further sections.

## Factors influencing pigment production

Pigment biosynthesis is greatly influenced by fermentation conditions, such as medium composition and process parameters. Sexual interactions have also been reported to increase the biosynthesis of the carotenoid β-carotene in some fungal species (e.g., *B. trispora*), where trisporic acids (substances with hormonal activity formed upon mating) were suggested to mediate the stimulatory effect [70]. Therefore, the various factors influencing pigmentation are interesting to consider in order to optimize the process of pigment production. The factors stimulating carotenoid production for microorganisms are summarized by Bhosale [46]. Overall, conditions that typically stress the cells, thus threatening cell growth, trigger carotenoid biosynthesis [8]. These typically include nutrition depletion (N and P) and certain levels of oxidative stress. Carotenoid accumulation usually occurs during the later stages of cultivation which also indicates the association of nutrient depletion with carotenoid synthesis [8]. This section addresses the effects of light, pH, nitrogen and carbon sources, temperature, co-factors, surface active agents, oxygen level, tricarboxylic acid intermediates and morphology in regards to *Neurospora* spp. carotenoid production, complemented with interesting discoveries on the regulation of pigment biosynthesis, particularly in *Fusarium* and *Monascus* species.

### Oxidative stress

To survive and compete in the environment, fungi produce enzymes and secondary metabolites with various activities. One such factor influencing this processes is oxidative stress [71].

The degree of oxidative stress has been shown to influence pigment production in filamentous fungi, indicating that these pigments are involved in the defense mechanism of the fungi. Numerous studies have reported observations [23, 37, 46, 63, 72–77] that support secondary protective roles of carotenoids against oxidative damage [63, 77]. For example, carotenoids can act as antioxidants against reactive oxygen species (ROS) [62] or alleviate cell membrane damage [63]. They have been proposed to act as antioxidative agents to extend the survival time of the fungi by synergistic effects with other antioxidants [62]. The ability of carotenoids to function as antioxidants may be the reason why dietary carotenoids have been shown to inhibit the onset of many diseases, such as cancer, in which ROS are thought to play a role [46]. It is the conjugated polyene chain of carotenoids that is able to chemically react as the quencher of singlet molecular oxygen [63], with varying efficiencies among carotenoids [72] depending on the structures beyond the polyene chain [63]. Astaxanthin, among other xanthophylls, have higher antioxidant activities than hydrocarbon carotenes, which make it advantageous for the fungi to synthesize. For example, the fungi synthesize astaxanthin at the expense of β-carotene under enhanced oxidative stress. This was confirmed when an increased synthesis of astaxanthin and less β-carotene by *Neurospora* was observed at increased oxidative stress levels (by addition of H_2_O_2_ and CuSO_4_) [63]. Other studies exposing carotenoid producing fungal strains to oxidants in order to increase synthesis of carotenoids have also been carried out [62, 78]. For example, in addition to the generation of reactive oxygen species (ROS) by respiration [8], exposure of filamentous fungi to paraquat (PQ) or hydrogen peroxide (H_2_O_2_) promote oxidative stress [79]. One study investigated the accumulation of the apocarotenoid neurosporaxanthin in *N. crassa*. They induced oxidative stress by exposing the fungi to high concentrations of oxygen and extracellular hydrogen peroxide (H_2_O_2_). In response to the elevated oxygen, the expression of genes encoding enzymes that are involved in the synthesis of carotenoids increased by a factor of five. On the other hand, H_2_O_2_ exposure resulted in a twofold increase in the accumulation of *al*-*1* mRNA [73]. Moreover, the addition of H_2_O_2_ to the fungi is suggested to work both as a pigmentation trigger and as an antimicrobial agent, making it an interesting factor to consider for further research, provided that the fungi do not consume it too fast [62].

Stressing the cells by inducing the generation of active oxygen molecules in order to enhance carotenoid production [46] can be done in different ways, which are proposed as secondary factors. These secondary factors are discussed below together with factors that are able to inhibit cell growth.

### Light

Carotenoids, in general, protect the fungi against UV-damage. The photoprotective function of *Neurospora* carotenoids are suggested to be due to their ability, as antioxidants, to quench damaging single molecular oxygen species generated by UV radiation [14]. Thus, light has been reported to influence carotenoid synthesis by inducing the enzymes involved in carotenoid synthesis [8]. The use of light as a factor in pigment production has been reported for *Monascus* spp. [27, 31], *Fusarium* spp. [80–82] and to some extent for *Neurospora* spp. [14, 23, 37, 63].

Carotenoids do not play a major physiological role in fungal cells, but they may have beneficial effects under certain adverse conditions, such as abnormal levels of UV light. This was corroborated by a study where albino mutants of carotenogenic fungi in *N. crassa* and others were compared with the counterparts of the same species with functional carotenoid synthesis. The lack of carotenoids showed no apparent phenotypic consequence on growth or morphology in laboratory cultures [37, 77]. *N. crassa* has been used as a model organism for photobiology research [83].

Stimulation of carotenoid synthesis by light is achieved at the transcriptional level, e.g., as seen by an increase in the mRNA levels of structural genes (*al*-*1, al*-*2* and *cao*-2) when neurosporaxanthin was produced by *N. crassa* upon irradiation [63]. The *al*-*3* gene in *N. crassa*, which is responsible for GGPP synthesis, has further been confirmed to be strongly regulated by light. The response was reported to be mediated by the photoreceptor and transcriptional factor called the white collar complex (WCC) comprised by the photoreceptors white collar (WC)-1 and its interacting partner WC-2 WC-1 is a blue light photoreceptor, responsible for the light-induced response. The protein contains a DNA-binding zinc-finger domain (that is able to bind specifically to the promoter of a blue light-regulated gene), and a PAS domain, called LOV (from “light, oxygen and voltage”). The WC-1 LOV domain binds to a light absorbing Flavin adenine dinucleotide (FAD) chromophore that convert light to mechanical energy. FAD displays a maximum absorption of light at 450 nm [84, 85], which explains the WC-1’s sensitivity to blue light. Thus, *Neurospora* perceives light through the WCC, which is activated by blue light and binds directly to light regulatory elements (LRE) in the promoters of their target genes to activate transcription [14, 86]. Consequently, light can be used to activate genes involved in the biosynthesis of pigments that have photoprotective functions [87]. Regarding *Fusarium* spp., carotenoid production is also stimulated by light, through the transcriptional induction of the structural genes *carRA, carB, carC and carT*, as the major regulatory. However, in *F. fujikuroi*, the main photoreceptor seemed to not be a White Collar protein, as found in e.g., *Neurospora* species [32, 35, 60]. Photocarotenogenesis in *F. fujikuroi* relies primarily on other putative photoreceptors, such as cryptochromes [60].

A study on carotenogenic fungi, including *N. crassa*, has shown that upon prolonged light exposure, there was a reduction in the level of transcription compared to that with exposure to light pulses [63]. Another report demonstrated that exposing *Neurospora* to light resulted in a rapid accumulation of colored carotenoids after 1 h, with increasing concentrations for up to 12 h. Aerobic conditions are required for these light responses to keep the photoreceptor in the right oxidation state [14]. The observation showing a cessation in the activation of gene transcription by light after a certain time is probably due the transient nature of light-induced transcription in *Neurospora.* Incubation in the dark is thus required before light responding transcription can be activated again. Regulation by light of pigment biosynthesis (photocarotenogenesis), is mediated, as other photoresponses in *Neurospora*, by the WCC. Upon extended illumination, the WCC-dependent transcript level decreases. The degree of ‘photoadaptation’ is by the fungus is modulated by the blue-light photoreceptor VVD. Strains with mutations in the *vvd* exhibited a sustained photoactivation of genes required for carotenogenesis [14, 87]. The amount and the intensity of light tolerated have been shown to vary with the strain, ranging 1000–5000 Lx [46]. Carotenoid photostimulation has also been reported to vary with different wavelengths for *Neurospora* spp. For instance, red light did not induce carotenoid biosynthesis in *Neurospora*, whereas wavelengths within 450–480 nm were shown to be effective [14]. Even though carotenogenesis in hyphal cells is induced by blue light and is lacking in mycelia grown in dark conditions, the synthesis of carotenoids has been found to be independent of light when coupled to conidiation and results in a pale pigmentation. [87]. Thus, *Neurospora* cultivated in the dark accumulate high amounts of the colorless precursor to carotenoids, phytoene, in their conidia. Illumination of these cultures grown in the dark induces transcription of enzymes responsible for the desaturation of phytoene, which leads to the formation of colored carotenoids [87]. More detailed descriptions of the regulation of carotenoid biosynthesis by light in *Neurospora* are very well illustrated by Díaz‐Sánchez Violeta et al. [35], Avalos Javier et al. [14], Olmedo Maria et al. [87] and Muñoz Victor et al. [88].

Further studies on light as a factor in pigmentation have found that *N. crassa* accumulates the orange carotenoid neurosporaxanthin after light exposure, with higher pigment content correlated to strains at lower latitudes. The increased carotenoid accumulation was suggested to serve as a second protection from harmful effects of UV radiation in *Neurospora* spp., since species that accumulate more carotenoids are more resistant to UV radiation [23, 37]. The ability of the fungi to accumulate different amounts of carotenoids [23] and the higher resistance to irradiation being connected to carotenoid production [72] strongly support light as a stimulating factor in pigment production. An attempt to increase carotenoid biosynthesis by *N. intermedia* in liquid substrate fermentation showed that fermentation with incubation for 3 days in the dark followed by 4 days under blue light was better than fermentation for 7 days in the dark. However, this difference in carotenoid production in different phases of life cycle has only been investigated in yeast (*Sporobolomyces ruberrimus* and *Cystofilobasidium*), and it showed a higher tolerance in the stationary phase compared to the exponential growth phase [89]. Direct information on such phenomenon for filamentous fungi is therefore still lacking.

### Nitrogen source

Limiting the availability of nitrogen is suggested to increase the concentration of total pigments in most strains of filamentous fungi [8], and this has been investigated mainly for *M. purpureus* [90–93] and to some extend in *Fusarium* [17].

The pigment variation depending on the nitrogen source is suggested to be influenced by the rate of amino acid metabolism [8] rather than directly by the nitrogen compound itself when the source is in the form of amino acids [94]. In this regard, amino acids that are metabolized slowly are favored since they induce nitrogen limitation to a greater extent. The carbon to nitrogen (C/N) ratio in the culture medium has also been proposed to be an important factor in addition to the nitrogen source. Generally, a high C/N ratio has been reported to promote carotenogenesis in many fungi as this condition limits the cell’s access to nitrogen [8]. The increased synthesis of pigments in limited nitrogen has been suggested to be related to a response mechanism to excess energy and carbon that cannot be used for protein synthesis or growth [8]. The balance between carbon and nitrogen sources at C/N 9:1 was reported to increase β-carotene production by *N. crassa*. The optimal ratio of carbon and nitrogen for the growth of *Neurospora* spp. in general has been reported to be within 7:1 and 15:1 [95], but its link to pigment production has not been investigated. Similar stimulation of carotenogenesis by a limiting nitrogen content has been reported for *Fusarium* spp. Rodríguez-Ortiz Roberto et al. [32] discovered that nitrogen exhaustion increased carotenoid production in both wild type and the carotenoid-overproducing mutants (*carS*, containing high levels of mRNA for the *car* genes) which had a high synthesis of carotenoids irrespective of illumination. The authors suggest regulatory connection between carotenoid biosynthesis and nitrogen controlled biosynthetic pathways in *Fusarium* [22]. The results indicate similarities in the regulation of nitrogen on carotenoid synthesis with *N. crassa*. Incubation of *N. crassa* under nitrogen starvation also increased the levels of the corresponding *al*-*1* and *al*-*2* independent on light [9].

Using a complex nitrogen source or the addition of individual amino acids have been shown to influence the number, type and excretion rates of different pigments that are formed [31, 42, 75, 96]. It has been suggested that the stimulating effect of amino acids on the production or liberation of pigments is caused by an increase in solubility because the derivatives linked to amino acids are more soluble than the original pigments [97]. It may be possible to obtain a desired number of pigment components by using a defined nitrogen source which provides specific amino acid moieties that can be incorporated into water-soluble extracellular pigments [97]. However, both positive and negative effects of different amino acids and their concentration limitations on pigment production have been reported. Furthermore, the mechanism behind these findings are still scarce in the literature and limited to only a few strains based on studies with *Monascus* spp. Such a phenomenon has not, to the best of our knowledge, been investigated for *Neurospora* spp.

### Carbon source

The carbon source is the most studied parameter regarding its effect on carotenogenesis in different fungi [77]. Singgih Marlia et al. [9] evaluated the carotenogenesis of *N. intermedia* in liquid fermentation. The highest production of carotenoids (24.31 µg/g spores) was achieved when 2% w/v maltose was used under aerobic conditions [9]. The other tested carbon sources included glucose, sucrose and maltose. Interestingly, a higher than 18 g/l glucose concentration was reported to reduced pigment production, perhaps due to respiro-fermentative metabolism [92]. These findings, however, oppose the previously mentioned report that suggested that an excess amount of energy and carbon limits the nitrogen source and stimulates pigmentation. The initial amount of the carbon source added to the cells has been investigated by Hailei et al. [41]. Their findings showed that it was the accumulation of glucose metabolites (glycerol, ethanol, organic acids and other substances), not the exhaustion of glucose, that played a major role [13, 41]. Another potential explanation for the relationship between stress due to carbon starvation and pigment production in *Neurospora* spp. could be the suppression of central carbon metabolism, which results in an increase in the acetyl-CoA pool available for other pathways, such as the mevalonate pathway (shown in part one in Figs. 4, 5, 6) [51]. The addition of ethanol as a carbon source has been reported to increase carotenoid production by growth inhibition, activation of oxidative metabolism and induction of HMG-CoA reductase [8], resulting in the production of lycopene and neurosporaxanthin in *N. crassa*.

The specific time of addition of different carbon sources has also been shown to influence carotenogenesis in *N. intermedia*, with a positive effect seen by addition at the middle of the log phase than at the beginning of the stationary phase. From this the authors concluded that the carotenoid content likely increased in the middle of log phase and then remained constant, which is typical for secondary metabolites [36]. However, general information regarding the time of addition on pigment biosynthesis is scarce in the literature and needs to be investigated further.

Although studies regarding the effect of carbon source is limited for *Neurospora*, more extensive research can be found for *Monascus* spp. [31, 77, 90, 98–101].

### pH

It has been previously reported that many kinds of fungi in submerged cultivation respond to more acidic pH conditions with the accumulation of pigments [102], probably due to the stress condition. Changes in pH during growth depend primarily on the nitrogen and carbon source in the medium [103]. Since research studies have supported the influence of pH on the pigment production by *Monascus* spp., it has been hypothesized as a factor to influence pigmentation in *N. intermedia* as well [31, 42, 44, 90, 92, 104]. The strong effect of pH on the biosynthesis of pigments in *Monascus* spp. has been proposed to be associated with changes in the activities of proteins. Changing the pH from neutral or slightly alkaline to more acidic has been shown to favor the cyclization of lycopene to β-carotene, and this has been applied in a patented fermentation process to improve lycopene yield [70]. It has also been noted that maximum pigment production is associated with the combined effect of pH and temperature in the culture medium. The effect was suggested to be associated with cellular growth, oxidation processes and metabolic flows regulated by molecules such as adenosine triphosphate (ATP). A change in the pH affects oxidation and reduction processes of molecules in the cell, thereby affecting the redox flux and the oxidative state of important energy molecules such as ATP, which has important roles in the cell metabolism. Changing the pH can therefore cause different metabolic pathways, substrate oxidation, and regulation of metabolic and osmotic processes, resulting in different end-products [104]. Nevertheless, the influence of pH on pigment production by *Neurospora* spp. is currently unexplored in the literature.

### Temperature

Changes in temperature affect cellular growth and metabolite production by enzymes, including those involved in carotenoid production in microorganisms [46, 77]. Thus, temperature is suggested to be a factor that may be utilized to regulate enzymatic processes connected to pigment production by the fungal cell [104]. The order of reactions in carotenoid biosynthesis can result in different carotenoids being produced depending on the temperature of growth [14, 47]. For example, at low temperature, the oxidative cleavage in the carotenoid pathway is favored over the cyclization reactions. This implies a competition between the enzymatic activities for the oxygenase and the cyclase for the same substrate (3,4-didehydro-lycopene, Fig. 4, 5, 6), and the conditions favoring one or the other depends on the growth temperature [14]. Cultivation at lower temperatures has also been reported to block the production of torulene and result in greater accumulation of β-carotene, probably due to changes in enzymatic activities [105]. Furthermore, illumination specifically at low temperatures increased the proportion of neurosporaxanthin in *Neurospora*. In general, the temperature influences not only the type of carotenoids produced but also the total carotenoid content in various carotenoid producing fungi [14, 17]. Studies have shown that exposing *Neurospora* mycelia to temperatures between 12 and 6 °C with illumination resulted in the highest response, although this phenomenon has not been described in other carotenoid-producing fungi [14]. This increase in carotenoid production below the optimal growth temperature is suggested to be an acclimating response compensating for the downregulation of metabolic processes and other changes, such as the fluidity of the cell membranes [46]. This strengthens the suggestion that carotenoids serve as a secondary protection for the cells.

### Co-factors

Co-factors such as metal ions and salts greatly affect fungal metabolism [106] and have been demonstrated to influence carotenoid synthesis [107]. Magnesium and calcium are considered macronutrients for filamentous fungi, whereas iron, manganese, zinc and copper are considered micronutrients [46]. It is probable that the effect of such co-factors on carotenogenesis occurs due to an activation or inhibition mechanism on specific carotenogenic enzymes [77], such as microbial desaturases [94]. Only a few attempts to address the biological roles of these cations in fungi have been reported [46]. One of the studies added up to 12 mM Magnesium ions which showed a stimulatory effect on pigmentation by *N. intermedia* [9, 31]. The magnesium ions were reported to stimulate the conversion of GGPP into phytoene (catalyzed by phytoene-synthase enzyme). Phytoene is desaturated to produce lycopene, which is a precursor of cyclic carotenoids in *N. intermedia* [9, 106]. Another study aimed to optimize red pigment biosynthesis by *M. purpureus* under solid-state fermentation and achieved higher concentrations of pigments by adding manganese rather than other macronutrients such as magnesium and calcium. This result was in line with findings from another study that cultivated *B. trispora* in trace amounts of manganese ions [46] or trace amounts of copper ions [108]. Manganese is a known cofactor for enzymes involved in carotenoid biosynthesis [109]. Manganese-dependent enzymes act on carotenoid production by influencing the concentration of cyclic AMP. cAMP has been shown to control a variety of functions in fungi [110, 111], which may include pigment production. One study investigated the role of exogenous cAMP on conidiation and carotenoid biosynthesis in *N. crassa* and found that it suppressed conidiation and lowered carotenoid synthesis [111]. However, the influence of abnormally low levels of cAMP is yet to be investigated. Nevertheless, another study reported that mutants of *N. crassa* with defects in the acyA gene coding for cAMP resulted in lower intracellular cAMP levels and contained more carotenoid pigments than wild-type cells [112]. García-Martínez et al. [113] also reported enhanced production of red pigments by *F. fujikuroi* with defects in the acyA gene [113]. A negative relationship between cAMP level and the accumulation of carotenoids in *N. crassa* [73, 114] has also been reported.

Another potential mechanism for how metal ions affect pigment production relates to the formation of active oxygen radicals (“Carotenoid biosynthesis” section) [94]. For example, pigment production was suggested to be induced by using ferrous ions to generate hydroxyl radicals (H_2_O_2_ + Fe^2+^ -> HO^−^ + HO*). Additionally, copper, zinc, lanthanum and cerium have been shown to have stimulatory effects on carotenoid yield via generation of active oxygen radicals [46]. However, negative effects on pigment production have also been reported for ferrous or cobalt ions, thereby indicating other effects of the metal ions in contributing to cell growth rather than promoting metabolic pathways [102]. Inhibition of carotenoid production was also shown in the case of adding manganese ions to a culture of *Xanthophyllomyces dendrorhous* containing oxygen radical generators. This effect was suggested to result from manganese acting as a scavenger or antioxidant by de-activating radicals, thereby resulting in lower stress to the fungi. The effect was thus suggested to be dependent on the concentration of manganese [46].

### Surface active agents

It has been reported that surface active agents (also known as surfactants), such as corn oil [90], Triton X-100 [13, 115], Tween-20, Tween-80 [42, 90], Span 20 [116] and olive oil, show positive effects on the metabolism of both intra- and extracellular pigments [42] depending on the strain and surfactant used. The amount of pigments that can be produced extra- vs. intracellularly varies with different cultivation factors and choice of strain. Secretion of extracellular pigments is favored over intracellular production since it requires less work to extract the pigments [115]. It has been reported that pigments access the aqueous environment by association with proteins or other polar compounds [117]. Surfactants are amphipathic substances that are able to adsorb onto surfaces of interfaces in dispersions and alter the interfacial free energy. Nonionic surfactants are able to form micelles in aqueous solutions and can be used as permeabilization agents for the secretion of hydrophobic intracellular pigments [115]. Transporting intracellular pigments to extracellular micelles will prevent pigment degradation and lower the intracellular pigment concentration, which otherwise decreases yield by product inhibition [13]. Surfactants are also suggested to act on cell membranes, increasing their permeability to release both enzymes and pigments into the medium. This was observed when Tween 80 was added to a culture with *Aspergillus amylovorus*, and it increased the pigment concentration in the medium by a factor of six [118]. It was also suggested that Triton X-100, which is able to solubilize membrane proteins, can increase the access of carotenoids in *Neurospora* to the aqueous environment by associating the pigments with membrane-bound enzymes [111]. In addition, Span 20 was suggested to affect β-carotene production by altering the fungal morphology [116] (“Carotenoid biosynthesis” section). However, when *N. crassa* was cultivated with the addition of Tween 40 (0.8%), carotene production was increased, but the carotene remained inside the cells [119]. Therefore, the way in which surfactants act on pigments is not yet fully understood since their effects are not consistent [118].

### Oxygen level

Because carotenogenesis is an aerobic process, oxygen supply is another important parameter of carotenogenesis [77, 120, 121]. Poor oxygen supply to the culture has been reported to decrease pigment production [104], and a critical dissolved oxygen tension (DOT) between 15 and 20% air saturation has been suggested for efficient carotenoid synthesis in fungi [8]. The crucial role of oxygen in pigment production is probably due to its ability to act as an electron acceptor during oxidative phosphorylation and as a substrate of monooxygenase. Oxidative phosphorylation and monooxygenation are involved in metabolite synthesis. In particular, monooxygenases are more active in secondary metabolism [31]. Thus, the relationship between oxygen supply and pigmentation is important to understand in order to achieve optimal production of pigments.

### Tricarboxylic acid intermediates

Intermediates of the tricarboxylic acid (TCA) cycle, such as succinate, citrate and malate, have been reported to stimulate carotenoid biosynthesis under aerobic conditions [46]. These intermediates form a carbon skeleton that can be used in pigment synthesis by different carotenoid-synthesizing species. Two potential explanations for the effect on pigment production by TCA cycle intermediates have been put forward in the literature. One explanation is that these intermediates are able to act specifically on some of the key enzymes involved in the isoprenoid biosynthesis pathway such as acetyl CoA-carboxylase, the starting substance for isoprenoid synthesis. The other explanation is that the effect is caused by the increasing pool of oxaloacetate from the added intermediates, since oxaloacetate is further decarboxylated to pyruvate leading to an increase of acetyl-CoA [46]. Likewise, high respiration rates and tricarboxylic acid (TCA) cycle activity are also associated with the production of large quantities of ROS [46], which could be another explanation for the increased pigment production when these intermediates are added.

Addition of citrate in *X. dendrorhous* and malate in *Blakeslea* and supplementation of 28 mM citrate to *Blakeslea trispora* have been reported to increase carotenoid production [8]. The degree of stimulation by the intermediates of the TCA cycle has also been reported to depend on the time during the cultivation when they were added to the medium [46]. However, these observations have not been investigated in ascomycetes, as far as we know.

### Morphology

Filamentous fungi can adopt diverse morphologies when cultivated in submerged cultures such as uniform and long filaments or entangled filaments in pellets or clumps. Morphology is also considered as a factor in pigmentation since it has been reported to influence the secretion of metabolites [13, 116]. A high fungal concentration with entangled mycelia or filaments results in a highly viscous suspension with non-Newtonian properties, which reduces the homogeneity of nutrition, temperature, oxygen, and other parameters. Growth in the form of dense pellets generates a less viscous medium with Newtonian properties, but the internal mass transfer rate is limited by pellet size and compactness. These changes in gas–liquid-mass transfer can thus affect the formation and secretion of products/metabolites [13]. One specific morphology that promotes pigmentation could be aimed for by adjusting stimulatory factors such as the cAMP level or fermentation conditions such as pH or oxygen supply rate. The influence of pH on morphology is explained in the study by Méndez et al. [104]. The study suggests that chemical or structural changes in the cell membrane induce morphology changes in the cell wall, which leads to an overproduction of pigments as a defense mechanism to regulate damage at the membrane level [104]. However, clearly established links between fungal morphology and pigmentation levels are still missing in the literature [104].

## Isolation, analysis and identification of carotenoids

### Extraction of carotenoids

In general, the isolation of intracellular carotenoids from filamentous fungi commonly involves a pretreatment step such as drying and/or cell disruption, an extraction step and a saponification step. Cell disruption is often necessary for intracellular carotenoids in order to increase the recovery. Many different methods for cell disruption have been suggested in the literature, including mechanical disruption (e.g., sonication, high-pressure homogenization, grinding, and bead-milling), and non-mechanical disruption (e.g., microwave assisted extraction, enzymatic hydrolysis, and ionic liquids) [122]. The preferred extraction procedure is based on a quick process that efficiently releases all the pigments from the matrix into the solution without altering them [56], while using environmental-friendly solvent(s), if possible. There is no standard method for carotenoid extraction from fungi, but based on previous studies, the general process involves the mixing of dried or wet biomass with organic solvents (liquid–liquid system), followed by mechanical disruption of the cells and subsequent centrifugation or filtration (pigment particles are approximately 1–2 µm). After filtration, the solid residue is re-extracted, and the process is repeated until the residue becomes pale. Three extractions are usually sufficient [56]. A saponification step is sometimes required for fat-rich biomasses in order to remove lipid contamination and to hydrolyze carotenoids found in ester or di-ester forms. The contaminating lipids may otherwise interfere with the chromatographic separation, identification and quantification of the carotenoids in later stages. When indispensable, saponification is most commonly carried out with 10% potassium hydroxide in methanol or ethanol, at temperatures below 60 °C to prevent carotenoid degradation [122, 123]. The carotenoid solution is then washed with water to remove the alkali. However, carotenoids with allylic hydroxyl and keto-groups, such as neurosporaxanthin, undergo oxidation in the presence of alkali and air. Saponification is not recommended for these carotenoids, or it must be carried out anaerobically. This procedure is further described by Schiedt Katharina et al. [124]. Finally, the total carotenoid content is commonly analyzed by measuring the maximum absorbance of the extracted pigments by spectral analysis using a spectrophotometer. The maximum absorbance for these pigments is in the range of 450–480 nm, depending on the type(s) of carotenoids present in the sample [14]. It should be noted that the position of the absorption maxima and the shape of the spectrum can vary by a few nanometers depending on the temperature and the presence of organic solvents [125].

There are some aspects to take into consideration when selecting the organic extraction solvent/solvents to use in order to optimize the solubility and stability of the carotenoids. Solvents with low boiling points are preferred since they are more easily removed through evaporation. If the fungal biomass contains large amounts of water, a water-miscible organic solvent (e.g., methanol or acetone) should be used for better solvent penetration [56]. Furthermore, the choice of solvent/solvents depends on the polarity of the carotenoids. Organic solvents commonly used to extract pigments include benzene, petroleum ether, hexane, acetone, chloroform, dimethyl ether, methanol, ethanol, [126], other alcohols, and ethyl lactate (described as a so-called green solvent) [127]. The organic solvents allowed in the EU for the extraction of natural food colorants are water, ethyl acetate, acetone, n-butanol, methanol, ethanol and hexane, while those allowed in the US are isopropanol, methanol, ethanol, hexane, and acetone [128]. Acetone is the most widely used solvent for carotenoid extraction since it is fairly harmless, inexpensive and readily available [56]. However, the polarity of the solvents and the pigments need to be considered. A better extraction is obtained when polar carotenoids, such as neurosporaxanthin, are dissolved in polar solvents, while less-polar carotenoids, such as lycopene, and γ- and β-carotene, dissolve better in non-polar solvents [129]. Therefore, if the fungal biomass contains a mixture of pigments, two- or three-stage extraction methods using organic solvents of different polarity may be required. A combination of different solvents has also been suggested to disrupt the cell and release intracellular pigments to a greater extent. For example, a mixture of petroleum ether, dimethyl sulfoxide and acetone was found to improve the extent of carotenoid recovery from *Rhodotorula glutinis* compared to the individual solvents [126]. Moreover, novel alternative methods that are more environmentally friendly compared to the conventional organic solvent extraction approaches are being developed. These include super- or sub-critical solvent extraction, switchable solvent extraction, wet extraction, and, more recently, ionic liquid-assisted extraction [122] and CO_2_ extraction [127]. These methods should be considered not only for environmental advantages but also for potential cost saving and increased efficiency [51, 122]. Research on “greener” extraction processes for fungal pigments should thus be further evaluated as an alternative to existing processes. The potential of emerging greener extraction systems for filamentous fungi pigments have been reviewed by Dufossé [15], Gong Mengyue et al. [122].

Only a few studies focusing on the isolation of carotenoids from *Neurospora* are available. One study analyzed the carotenoids produced by *N. intermedia* in liquid fermentation. Disruption and solubilization of the carotenoids present in the biomass were made using acetone as the extraction solvent and sonication for cell disruption. The total carotenoid content was determined to be 0.8 mg/g spores by measuring their absorbance by UV/Vis spectrophotometer at 480 nm [9]. Another study [36] utilized a two-stage extraction. Methanol was used as the first extraction solvent and acetone was applied as the second extraction solvent on dry mycelia, at 60 and 50 °C, respectively. The resulting methanol/acetone fraction containing the extracted carotenoids was collected, centrifuged and the spectral absorption at 470 nm was determined [36]. In another study, extraction of carotenoids from conidia of *N. crassa* was performed at an elevated temperature but with ethanol as the solvent, and the carotenoid content was estimated by measuring the absorbance at 475 nm [112]. The results obtained were expressed in terms of units of absorbance (U/g dry cell mass), a value proportional to pigment concentration [92]. The *λ*_max_ values and solvents used for extraction of various carotenoid pigments from *Neurospora* spp. are cited in Table 1.

### Purification, characterization and identification of carotenoids

Since *N. intermedia* and most other filamentous fungi accumulate a complex mixture of pigments, the next step includes techniques to purify and quantify the pigments that are present in the mixture. A purification step is needed if the extracted carotenoids are to be used, for example, in food and cosmetic applications, or for certain quantification methods [51]. Purification and quantification of the extracted pigments are based on chromatographic and spectroscopic properties, and chemical tests. Column chromatography, thin-layer chromatography (TLC), ultraviolet–visible spectrometry, and high-performance liquid chromatography (HPLC) with online photodiode array detection are commonly used to separate, identify and quantify carotenoids [130]. HPLC systems combined with photodiode array detector (PDAD) separates and identify carotenoids found in fungi, in view of their high sensitivity, reproducibility and short analysis time, while minimizing isomerization and oxidation of unsaturated carotenoids [131]. This technique relies on the characteristic differences in wavelength maxima for each carotenoid and the spectral fine structure. Larger numbers of conjugated double bonds in the carotenoid structure will shift the wavelength maxima (*λ*_max_) towards longer wavelengths The long conjugated polyene system, which makes the *trans* isomers linear and rigid molecules, is an important property for the interaction of carotenoids with the stationary phase in HPLC and for their absorbance of light in the visible region at 400–550 nm (Figs. 4, 5, 6). Generally, carotenoids absorb light maximally at three wavelengths (three-peak spectrum) where most other substances do not absorb. This property can be utilized when carotenoids are to be identified in complex mixtures [56]. In a previous study, measurements of the amount of β-carotene produced from *N. intermedia* have been carried out by HPLC using a C_18_ column with acetonitrile: methanol: 2-propanol (85:10:5) as the mobile phase. Detection of the carotene was carried out with a UV/Vis detector at 450 nm and the compound was compared with β-carotene standards [9].

Characterization and identification of carotenoids are described in more detail by Gross [123].

## Industrial applications and challenges

### Potential application areas and present status of the market

The pigment industry, mainly the food industry, is looking for potential sources and uses of natural-origin pigments without harmful environmental and health-related side effects, in addition to new colorants with improved functionality and a constant supply of raw materials from cheap and reliable sources. Consequently, the application areas of naturally derived pigments from *N. intermedia* are broad and have a bright future on the market if successful at an industrial scale.

Potential applications of carotenoids include their use in animal feed to improve the nutritional profile and to enhance the appearance of poultry skin, salmon meat and shade of egg yolks, the colors of which are determined by the animals’ diet. For instance, the apocarotenoid astaxanthin, mostly produced from microalga and bacteria, has an orange-to-reddish color and is used currently as feed-additives in aquaculture due to its health-promoting properties. This compound provides the typical orange pigments to salmonids, lobsters and trout in addition to being used as a color enhancer in the diets of chickens to improve the color of egg yolks to meet consumers’ expectations [132]. Some natural-origin pigments are also used as intermediates for dyestuff for textiles and biodegradable polymers [133]. Furthermore, they can replace synthetic pigments used in food, drugs, cosmetics and healthcare products. Extraction of pigments from plants has been the predominant source of natural pigments thus far, but the use of these pigments is limited by their available quantities [134], irregularity of harvests, land use and labor-intensive characteristics [8]. Microbial pigments, on the other hand, have shown greater stability against external stress such as light, pH and temperature and have high water solubility [6, 104] compared to pigments from plant sources [7]. Microbial pigment production is also an environmental friendly method compared to synthetic pigment production [8]. To date, more than 600 carotenoids have been found to be produced by carotenogenic microorganisms, but only astaxanthin and β-carotene are commercially produced by microbial fermentation. In 2010, synthetic colorants accounted for 40% of the colorants available in the market, whereas natural-derived colorants and nature-identical colorants (i.e., man-made pigments which are also found in nature [135]) accounted for 31 and 29% of the market, respectively. However, due to the advantageous associated with fermentation-derived natural pigments from microbial sources mentioned above [9] these may be a promising alternative that could tackle some of the current problems. According to Mapari et al. [7], products with natural colorants are expected to replace synthetic colorants in the future.

Microalgae are one alternative source of colorants, but their low productivity limits their use on a commercial scale. The carotenoids in fungi (include β-carotene, γ-carotene, torulene and their hydroxyl- and keto-derivatives [136]) grant industrial interest. These natural-origin pigments could be exploited for their antioxidant, provitamin A activity [137] and beneficial effects on the onset of many diseases [13, 65]. Strains of *Basidiomycetous* fungi have been used for coloring silk and wool but are limited by their difficulty to grow under laboratory conditions and are therefore not suitable for production on an industrial scale. On the other hand, ascomycetes have been traditionally used in different parts of the world for hundreds of years for food coloring, and they can be easily cultivated to give high yields [5]. The interest in these pigments is also growing because many ascomycetes are known to secrete pigments with improved functionality (e.g., with anticancer properties) [13]. Particular attention has been given on *Monascus* pigments which have been shown to possess heat and pH stability during food processing [12].

Another potential pigment producing fungi with biotechnological applications are species of *Fusarium* that synthesize e.g., bikaverins (red pigment) [16] and carotenoids [32]. Research projects using *Fusarium sporotrichioides* for production of β-carotene and Lycopene [15, 138] and *F. fujikuroi* for improved neurosporaxanthin [40] and β-carotene [139] production suggest high potential in the field. Bikaverin is also biotechnologically relevant due to its selective toxic effect against tumoral cells [140] and its antibiotic activity against some phytopathogenic fungi and protozoa [141, 142]. One drawback with *Fusarium* spp. is, depending on the growth conditions, its potential of co-production of mycotoxins such as trichothecenes, fusarins, and zearalenone [10, 22]. This can be overcome by carefully regulated cultivation conditions or genetically modifying the fungi. *Fusarium venenatum* is known for its myco-protein production used for human consumption [11]. The potential industrial interest may increase by genetically modify the fungus to produce carotenoids that would add health value to the myco-protein product [143].

Even though most fermentative food-grade pigments from filamentous fungi are at a development or research stage [13], there are a few that already exist in the market. These include Arpink red™ (now Natural Red™) from *Penicillium oxalicum* (manufactured from Ascolor Biotech) [5, 65, 83], which has received a 2-year approval by the EU to be used as a food additive in the Czech Republic from 2004 to 2006 (current status of approval unknown) [4], riboflavin from *Ashbya gossypii* [97, 144], lycopene and β-carotene from *B. trispora* (produced by Gist-Brocades, now DSM; approved in 2000 by the EU Scientific Committee on Food Safety) [46, 47, 97] and previously mentioned *Monascus* pigments [66, 97]. The industrial production and use of β-carotene and lycopene from *B. trispora* as food colorant have been approved by the European Commission in 2000 and 2006, respectively [51]. As an example, β-carotene has been developed to yield up to 30 mg/g dry mass or approximately 3 g/l of culture in a submerged fermentation process [2]. Production of β-carotene and lycopene at larger scales (25 m^3^) under normal fermentation conditions are expected in the near future [46]. Regarding the industrial applications for *Monascus* pigments, the most common species used are *M. purpureus*, *M. pilosus* and *M. ruber* [13, 145] due to their production of orange, red and yellow pigments [29, 30] that are used as natural colorants for making red rice, red soybean cheese, wine, marine and meat products [4, 31]. Their yellow and orange polyketide pigments have been commercially produced and legally used as food colorants in the Southeast Asia for more than a thousand years [2].

Until now, β-carotene has been the most economically important carotenoid with a market share of US$261 million in 2010, followed by astaxanthin and lutein [77] with global market shares of US$252 million (2010) and US$60 million (2011), respectively. However, only 2% β-carotene is derived from natural sources [122], mainly from palm oil and to some extent fermentatively produced by *B. trispora* [12]. β-Carotene is the most well-known dietary source of provitamin A and believed to be the most important carotenoid in human nutrition [8]. It has been used in health and food products and to enhance the color and appearance of fish, birds, processed meats and tomato ketchup among other applications. Lycopene, produced mainly from tomatoes [12], has many applications within the food industry due to its red color and strong antioxidant activity (strongest among the carotenoids) [8], and it is used mainly as a nutritional supplement or active ingredient in cosmetic products [146]. It has also been suggested to have anti-carcinogenic properties [147]. Additional carotenoid compounds found in *N. intermedia* and their industrial applications can be found in Table 2.

### Challenges for large-scale pigment production and future prospective

Pigments used for food colorants need to be approved by a regulatory agency and the most important factor in their consideration is safety. Consequently, there are many limitations and legislations covering this issue [13]. The approval (2000) by the EU for the use of filamentous fungal carotenoids as food colorants has renewed the interest on the use of fungi as carotenoid producers. The United States regulation on the use of pigments in food and conditions for their use is outlined in Code of Federal Regulations, Title 21 (21CRF), which is also followed by several other countries, while the Australian and Japanese legislations are also used in a number of other Asian countries. The legal use of filamentous fungi in different parts of the world varies with local and traditional usage of colorants [2]. For example, *Monascus* pigments have been used for more than thousands of years in China, Japan, and other Southeast Asian countries but are not permitted as colorants in the European Union and in the United States [97, 145]. A legislation by the European Parliament reinforced the need for alterative “green” carotenoids by requesting that foods containing synthetic colorants, including quinolone yellow and tartrazine, require a label stating “may have an adverse effect on activity and attention in children” [5].

Other important desired features include a high pigment yield, ability to dissolve in water and the production of stable pigments [7]. Furthermore, the potential production of other secondary metabolites such as mycotoxins [30, 120], the kinds of carotenoids produced, [77] and whether the pigments are produced extra- or intracellularly are important factors to consider [97]. The production of pigments will also depend on consumer approval and the production costs required to bring the product to the market [7].

Regarding the pigment yield, there are generally two natural ways in which the amount of pigments can be increased by improving the fungal growth, or by increasing the cellular accumulation of pigments [148, 149]. The problem, however, lies with the difficulty in increasing both the biomass and the pigment production, which would be optimal for industrial production. Biomass and pigment yields are suggested to be negatively correlated [104]. An increase in the biomass yield is connected to the abundance of nutrients in the medium whereas pigment production seems to be increased under nutrient-poor conditions and external stresses. Therefore, the relationship between biomass and pigment production needs to be fully elucidated in order to optimize and control pigment production. One way to control pigmentation in fungi is to use recombinant DNA techniques. Genetic modifications have been previously applied to alter the activity of enzymes involved in carotenoid biosynthesis [46, 148–151]. All carotenoids share a common precursor, which can be utilized to manipulate the carotenoid biosynthetic pathway by combining biosynthetic genes and produce a much wider range of carotenoids [8]. However, genetic modification lowers the acceptance for the use of the produced pigments in food and feed industries [42].

Another challenge involves the differences in bioavailability and absorption rates between different types of pigments. Bioavailability refers to the amount of pigments absorbed in the body (to become available for physiological functions or storage) and is thus desired if the pigments are to be used in the feed industry, for instance. A high absorption rate, in this context, relates to the release of pigments to the product matrix, for example, in coloring a food product. Naturally, the food matrix itself will affect the absorption and the release of pigments [137]. Hence, the choice of pigment is important, as different pigments vary in bioavailability and absorption to the animal, human and food product matrices [127]. There are still limited data about differences in bioavailability and absorption between various natural-origin pigments, but, in general, it seems that more polar carotenoids (xanthophylls) are absorbed more efficiently than hydrocarbon carotenoids (carotenes) [152, 153].

The few strains of filamentous fungi that already exist in the market still have some challenges that need to be addressed to be able to compete with other pigment sources on the market. For example, the carotenoids produced from *B. trispora* exhibit good pH stability in most foods but are easily oxidized [12], although this can be countered by addition of antioxidants [154]. Challenges for the commercial production of pigments produced from *Monascus* include the pigments’ low water solubility, sensitivity to heat, instability at pH 2–10, and fading color intensity with light. To make the pigments more water-soluble, methods have been developed to substitute the replaceable oxygen in the pigment structure with nitrogen from the amino group of various compounds such as proteins, peptides and amino acids, and some patents have addressed these challenges [144]. Furthermore, when using *Monascus* for the production of pigments for feed and food applications, another problem is the co-production of citrinin, an azaphilone with nephrotoxic and hepatotoxic properties. Safety concerns regarding citrinin, a compound classified as a potential human carcinogen [30, 120], limits the use of *Monascus* pigments although some edible *Monascus* spp., such as *M. purpureus*, have been used for the production of red fermented rice for over a thousand years in Asian countries, [143]. This has prevented the approval of *Monascus* pigments as food colorants in the European Union (EU) and the United States (US) [5]. Similarly, *P. oxalicum* also produces the yellow toxic pigment, secalonic acid D [5]. On the other hand, *N.* *intermedia*, isolated in 1842, has neither been observed to cause diseases in plants or animals nor to produce dangerous secondary metabolites (e.g., mycotoxins), and thus, it has been extensively used in the food and beverage industry [19]. This edible fungus has traditionally been used for the production of the Indonesian fermented food oncom [6], contributing to the characteristic orange color of the dish. Hence, *N.* *intermedia* is generally recognized as safe (GRAS) [19]. Moreover, it is closely related to *N. crassa*, a very well-evaluated model organism [155]. Nevertheless, among the *Neurospora* spp., only *N. crassa* has so far been evaluated for industrial production of pigments in a research project [130].

Since most fungi produce a mixture of pigments, another challenge is to be able to direct the pigment production towards one specific colored dye in the future. The amount of different pigments can be adjusted by altering the substrates, the operational conditions (pH, dissolved oxygen, temperature) and fermentation mode [solid state fermentation (SSF) or submerged fermentation (SmF)].

The choice between solid state fermentation (SSF) and submerged fermentation (SmF) has been discussed extensively in previous reviews regarding pigment production by filamentous fungi, especially for fermentation with *Monascus* spp. [26]. SSF is generally the process for the production of *Monascus* spp. pigments and is known to provide more pigments than SmF. However, research on the use of SmF has demonstrated easier handling, lower production costs, shorter cultivation times and higher product quality [31, 156]. Furthermore, there are several SmF techniques that can be used, such as batch or continuous mode, in order to achieve optimal pigment yields [13]. To date, most of the industrial production of carotenoids by filamentous fungi use SmF, while the use of SSF is still in the exploratory stage [107]. SmF may generate a higher pigmentation because oxygen and light are required for maximum pigmentation. To the best of our knowledge, studies focusing on pigmentation by *N. intermedia* have not addressed different fermentation strategies. Therefore, it remains to be seen if SSF will compete with SmF in the future. Furthermore, if pigment production is to be incorporated in an already existing biorefinery, the impeller design and configuration or the aeration rate in the stirred-tank and airlift bioreactors should be optimized for optimal gas exchange in order to increase pigmentation.

### Pigments as a value-added product within biorefineries

To produce cost-effective pigments with less environmental impacts, the choice of substrate is of great importance. Using residues from industrial processes to create new products is certainly something worth aspiring to.

Substrates commonly used for solid state fermentation of *Neurospora* spp. have been based on waste products of plants or cereals that are rich in amino-acids and carbohydrates [106]. β-Carotene production by *N. crassa* has been investigated in a study by Thomson ISI [39] where the fungi were grown on various residues with the aim to produce carotenoid rich feed. A mixture of tapioca by-product (60%) and tofu waste (40%) resulted in the highest β-carotene content (295 µg/g) [95]. *N. intermedia* was also suggested to produce high concentrations of carotenoids when the solid waste from tofu production was used as substrate [37]. Similarly, when *Neurospora* spp. were grown on 80% sago waste and 20% tofu waste, 246 µg/g of β-carotene was produced [95]. Studies on different waste sources as substrates for fermentation-derived pigment production by *Neurospora* spp. are interesting from an economical and environmental point of view and thus need to be investigated further.

**Fig. 7:**
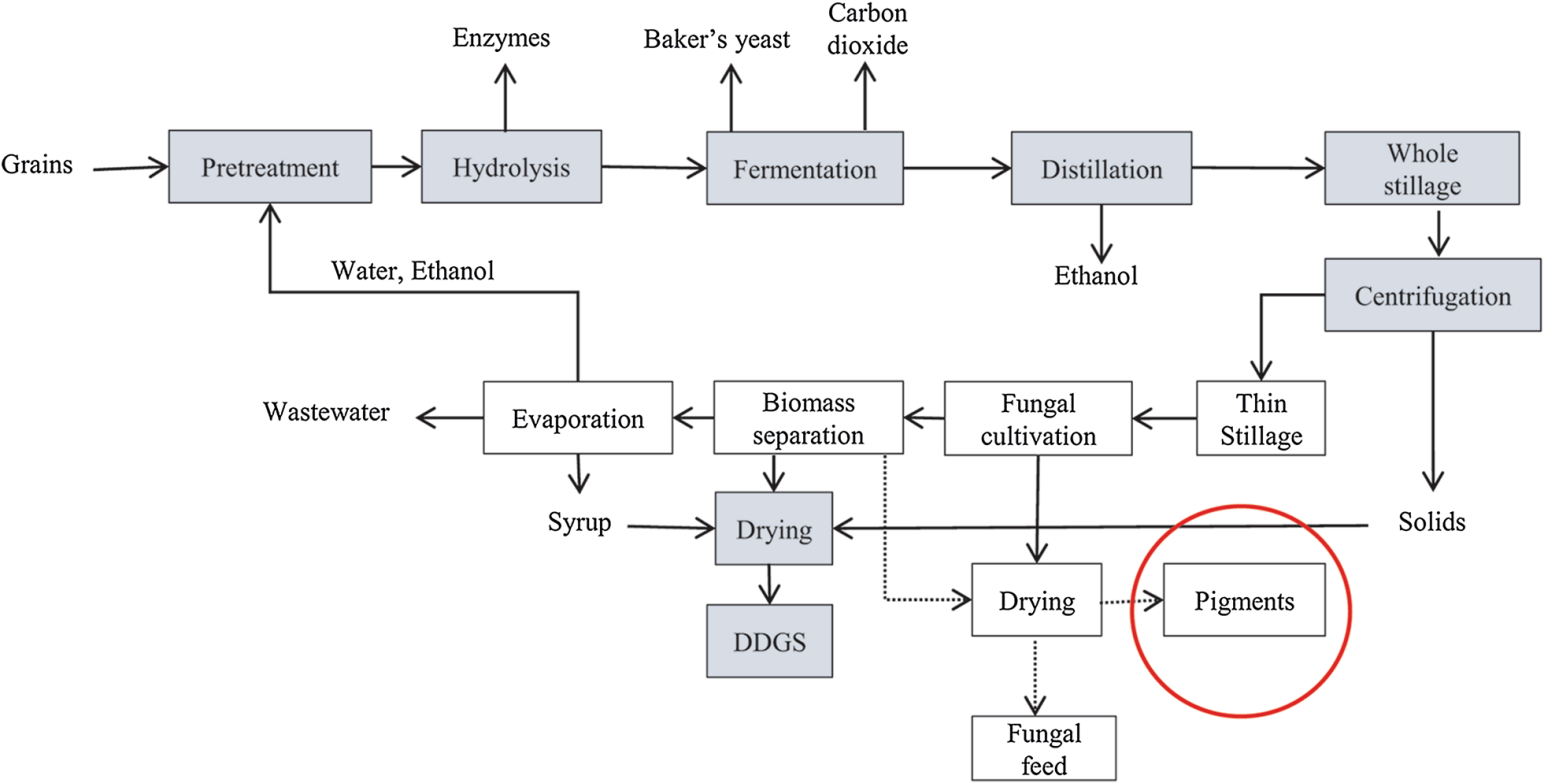
Schematic process scheme of the main starch-based bioethanol process stages leading to the production of ethanol, DDGS and CO_2_. Adapted from Ferreira et al. [120], and suggested production of pigments represented in *dashed lines* using filamentous fungi

*Neurospora intermedia* is under evaluation [1] as a second biocatalyst in the industrial production of bioethanol for yielding ethanol and fungal biomass from fermentation left-overs [120]. The production of pigment-containing biomass would open the possibility for their use in salmon or chicken feed, which would be in line with the studies aimed to improve the nutrient content in poultry feed [95]. The pigment-containing biomass could potentially be used for human consumption as well. The process has been scaled up to 80 m^3^ and further expansion is expected in the near future. Therefore, the successful control of pigment production by *N. intermedia* could reinforce the biorefinery character of the 1st-generation ethanol plants via the production of value-added products. Figure 7 shows an example of how pigment production can be integrated into an already existing biorefinery plant by using the residues as substrates.

## Future prospects

Due to increased health and environmental concerns along with tougher regulations regarding the use of synthetic pigments, intensive research is being carried out to find sources of natural-origin pigments. The use of filamentous fungi as an industrial source of biomass and value-added products such as organic acids, enzymes and pigments is already a reality.

Research focusing on *N. intermedia* is also expected to increase due to its potential ability to grow on more economically and environmentally friendly substrates from process leftovers. This also entails the production of value-added products, such as pigments, by process diversification. The potential use of the fungus for this purpose is further strengthened by its pigment production without co-production of any mycotoxins. Therefore, the food-grade ability of the fungus will tentatively aid in public acceptance regarding the use of its pigments for food and feed applications.

Over the years, several studies have focused on factors that stimulate pigment production in filamentous fungi. However, these studies have been superficial and have only briefly considered the optimization of pigment production at a larger scale and considered the circumstances that lead to pigment production as well as the potential regulation towards different color dyes. Moreover, the correlations between the factors are not entirely clear either. Hence, the substrate, cultivation conditions and bioreactor design need to be developed and optimized in order to control the process towards pigments and other desired products. From an industrial point of view, it would be interesting to investigate if pigment optimization is correlated with other valuable features, such as favorable morphological structures (pellets) and/or production of other metabolites. To date, the use of *N. intermedia* for pigment production on an industrial scale has not yet been explored.

As the research goes on, knowledge gaps are filled, hypotheses are proven and production processes are verified, it remains to be seen if pigment production using this fungus will be able to compete with other processes where plants or other organisms, such as algae, are used. Therefore, optimization but also comprehensive characterization of pigments produced by *N. intermedia* will naturally play a crucial role in this regard.

## Conclusions

Natural-origin pigments, such as carotenoids produced from filamentous fungi, are valuable bioactive compounds with an increasing market demand to replace chemically synthesized pigments. However, there is a need to improve fermentation strategies, control pigment production and decrease production costs in order to compete with synthetic pigments. Carotenoid production in fungi seem to be triggered by environmental stresses, and these conditions can be achieved by regulation of the fermentation process. *N. intermedia* has been foreseen to be a very promising candidate for pigment production due to its color, absence of mycotoxin production, and versatility regarding substrates that it can grow on. The production of pigments adds great potential for biorefineries in which filamentous fungi can be core biocatalysts to convert by-products into several value-added products. By filling the gap in knowledge regarding the production of pigments by *N. intermedia*, the process of pigment-production might be accomplished by research efforts to include the fungus in 1st-generation ethanol plants.
